# Training and Assessing Model for the Ability of Community Health Volunteers in Anthropometric Measurement Using the Rasch Stacking and Racking Analyses

**DOI:** 10.1155/2021/5515712

**Published:** 2021-09-21

**Authors:** Deni Kurniadi Sunjaya, Dewi Marhaeni Diah Herawati, Noormarina Indraswari, Ginna Megawati, Bambang Sumintono

**Affiliations:** ^1^Department of Public Health, Division of Public Health, Faculty of Medicine, Universitas Padjadjaran, Bandung, Indonesia; ^2^Departement of Public Health, Division of Medical Nutrition, Faculty of Medicine, Universitas Padjadjaran, Bandung, Indonesia; ^3^Departement of Public Health, Division of Epidemiology, Faculty of Medicine, Universitas Padjadjaran, Bandung, Indonesia; ^4^Faculty of Education, University Malaya, Kuala Lumpur, Malaysia

## Abstract

**Background:**

Inappropriate anthropometric measurements of infants and toddlers lead to a misclassification in nutritional status and loss of important interventions. Considering the practice conducted in this program within a country, its impact on millions of children must be considered. This study assesses the ability of community health volunteers (CHVs) before and after anthropometric training on infants and toddlers. Methods. This study used a quantitative approach with a quasiexperimental and pretest-posttest design. The pre- and posttraining assessments of CHVs were conducted by standardized trainers using instruments developed according to WHO standards. There were 11 and 13 statement items for infants' and toddlers' indicators of assessment in anthropometric measurements, respectively. The result of the assessment was then analyzed using Rasch modeling with stacking and racking data analysis techniques.

**Results:**

The CHVs' skills before training were far from adequate. Although widely varied, all trainees improved their abilities. Stacking analysis showed that the skills of all CHVs in measuring infants and toddlers increased by 2.68 and 3.34 logits (*p* < 0.01), respectively. Racking analysis showed a decrease in the perceived difficulty of all items by 2.61 and 3.07 logits for infant and toddler measurements, respectively (*p* < 0.01). The results of the racking analysis showed that the difficulty in measuring the anthropometrics of infants decreased more than that of toddlers.

**Conclusions:**

CHVs' capacity to monitor child growth must be refreshed regularly. Standardized and proper training and assessment were developed to make CHVs reliable in taking anthropometric measurements of infants and toddlers.

## 1. Introduction

Indonesia, a country in the Asia Pacific, has a high prevalence of stunting (36.4%) after Nepal (37.4%), Lao PDR (43.8%), Pakistan (45%), Yemen (46.5%), and Papua New Guinea (49.5%) [1]. According to the results of the Basic Health Research conducted by the Ministry of Health in 2018, the prevalence of stunting in Indonesia has declined to 30.8% [2].

The Indonesian government made several efforts to reduce the prevalence of stunting with nutrition-specific and -sensitive interventions. One of the nutrition-specific interventions is the growth monitoring (GM) program, where community health volunteers (CHVs) conduct anthropometric measurements in Integrated Healthcare Post (IHP) [3]. According to the World Health Organization, growth assessment requires anthropometric measurements and the results are compared to reference standards [4]. The anthropometric measurement process is critical because measurement accuracy is an essential indicator to obtain accurate and high-quality data; moreover, errors in these measurements can affect interpretation [5].

The UNICEF promotes GM, which is an activity in child care conducted universally [6]. The GM graph has been widely used by various countries globally [6]. This is a cost-effective program for reducing the prevalence of stunting [7]. Currently, many scholars doubt the effectiveness of implementing GM alone [8], considering factors such as CHVs failing to provide education to parents whose children have nutritional status problems; hence, they believe conducting growth monitoring and promotion (GMP) is better [9, 10].

CHVs in Indonesia were assigned to run child measurement tasks at IHP after receiving training from the community health centers (CHCs). Still, there is no systematic evaluation to assess whether CHVs have a proper skillset. GMP is a preventive activity that employs GM (i.e., measuring and monitoring growth and development) to facilitate communication and interaction between CHVs and parents/caregivers to prompt adequate actions that encourage children's growth and development. GMP is essential in child care as it measures, records, and interprets growth to provide counseling, therapy, and follow-up [11].

Indonesia has 9,993 CHCs, located in a subdistrict. There are 283,370 IHPs attached to the CHCs, and approximately 1,416,850 CHVs are present across all districts and cities [12]. Moreover, 89% rural areas and 11% urban areas constitute Indonesia. Several aspects, such as capacity, educational level, and CHVs' training in urban areas, are better than those in rural areas [13]. However, there is not much information on the difference in the capacity of CHVs in rural and urban areas. CHVs' ability is a crucial factor in providing services in CHCs, especially in conducting anthropometric measurements.

GM activities are conducted by CHVs volunteering to provide health services in the community. All CHVs are housewives who agreed to become CHVs. On average, they have worked as CHVs for approximately three years. Before conducting GM, all CHVs were trained by CHCs; unfortunately, this training system does not have any form of evaluation to assess, in detail, the ability and understanding of CHVs in conducting anthropometric practices for infants and toddlers. Some CHVs did not receive any training because they replaced other CHVs who had dropped out. Furthermore, most CHC officers who provide these training are yet to receive standardized training.

CHVs have diverse socioeconomic backgrounds and educational levels, so their ability to perform anthropometric measurements varies widely. Monthly GM reports by CHVs are used by CHCs and the health office at district and provincial levels as routine data to be reported to the Ministry of Health. CHC is responsible for ensuring the quality and capacity of CHVs. Besides, CHC is also formally responsible for monitoring the nutritional status of infants aged 0–2 years and toddlers aged >2–5 years, under their working area.

Inappropriate anthropometric measurements of infants and toddlers will cause misclassification in nutritional status, planned interventions, and referral and will divert parents' attention [14]. Misclassified nutritional status leads to inappropriate intervention. Infants who are *severely stunted* but are misclassified as *stunted* will receive inappropriate intervention. Misclassification also affects the referral process. For example, a baby with severe stunting may require monitoring by a pediatrician; however, because the nutritional status was misclassified, the baby was not consulted. Additionally, if a stunted infant is incorrectly classified as normal, parents may have false beliefs. Consequentially, they may treat their children without regard to nutrition and general health.

Anthropometric measurements of toddlers conducted periodically in a village population can portray growth, nutritional status, and health. Moreover, it can be a reliable tool for evaluating a village's health and social problems [15, 16]. Additionally, anthropometric measurements are easier to determine nutritional status than other advanced methods [17]. Measurements used for growth indicators are weight, length, height, head circumference, and mid-upper arm circumference (MUAC).

Standardized training is a prerequisite for reliable CHVs who would be taking anthropometric measurements of infants and toddlers. According to the WHO, standardized training must achieve high precision in measurements [18]. CHVs highly need regular training to perform measurements with high precision. According to Arroyo et al., periodic training is even required by dietitians to minimize errors when performing anthropometric measurements [19]. According to Mwangome et al., CHVs can be trained to measure MUAC, length, and weight of infants under six months of age to yield reliable and accurate results [20].

Inappropriate anthropometric measurements will cause misclassification and misdiagnosis of nutritional status, resulting in a loss of proper interventions. Considering that the practice is conducted as a program within a country, we can imagine the impact on millions of children and generations. This study evaluated CHV's ability to perform anthropometric measurements after being trained. The training and assessment model will be advocated subsequently to the government.

## 2. Materials and Methods

### 2.1. Study Design

This study used a quantitative approach with a quasiexperiment pretest-posttest design. CHVs/volunteers of IHP were given anthropometric training, such as measuring body height, body weight, upper arm circumference, and head circumference of infants and toddlers, and were assessed in practicing anthropometric skills. The evaluation before and after training was conducted and observed using standardized instruments.

Rasch analysis with stacking and racking data techniques was used in this study [21–23]. To date, many studies have been conducted to evaluate the effects of anthropometric training in CHV's capacity. Nevertheless, the analyses emphasized more on group-centered statistics and could not give analyses on CHV's individual level and to individual's items difficulty. Alternatively, Rasch analysis with stacking and racking data techniques can differentiate CHV's individual ability before and after training. Moreover, using this technique, the aspects of anthropometric measurements that were the most difficult to understand by CHV could be identified and corrected.

### 2.2. Ethical Approval

This study fulfilled Helsinki's Declaration and received ethical clearance from the Ethics Committee of the Faculty of Medicine Universitas Padjadjaran, No. 1481/UN6.KEP/EC/2019. Informed consent was obtained from all subjects before participating in this study.

### 2.3. Location and Subject

The study site was in Cirebon District of West Java Province, one of the stunting loci determined by the Ministry of Health of the Republic Indonesia. The anthropometric training was conducted in 10 rural subdistricts with stunting problems. Three of the 10 subdistricts were randomly selected for data collection in this study. There were 41 female CHVs from three subdistricts who participated in this study. The minimum age for a CHV is 19 years, with at least primary school graduation. Demographic data of this study were a mean age of 40 years and three years of experience for most CHVs; there were five (12%) of them who graduated from a primary school, and the rest (36 or 88%) graduated from junior high school and above.

### 2.4. Training and Assessment

There were 10 trainers as facilitators and 20 observers in this study to provide one-day training to 41 CHVs, divided into 2 classes. First, CHVs were trained using standard materials from the WHO and the Ministry of Health [24, 25] on anthropometric measurements for infants (0–2 years) and toddlers (>2–5 years). Before the training, CHVs were informed that there would be a pretest and a posttest. During the pretest, 41 CHVs were asked to measure infants and toddlers' height, weight, upper arm circumference, and head circumference. Observers then assessed their anthropometric skills.

After pretest, the facilitators delivered material on the correct measurement method according to the standard. They presented the correct measurement that should be carried out, what errors often occur, and how to avoid these errors. The facilitator demonstrates/practices how to take the correct measurements and then trains the CHVs to do it by themselves and make corrections if needed. Thereafter, the CHV was asked to perform anthropometric measurements using model dolls for real babies and toddlers. During the anthropometric practice, the trainer provides corrections and feedback where needed, for instance, having missed a step and used the wrong equipment. Upon completion, all CHVs must demonstrate their anthropometric skills to be assessed by the observers.

### 2.5. Instruments

The instruments used for evaluation in this study were standardized to measure anthropometric skills for infants and toddlers. The instruments were developed based on theories on anthropometric measurement and the volunteers' skill capacity [26]. Infant's and toddler's anthropometric skill indicators were grouped in three dimensions, comprising 11 and 13 items. Each item had three ratings to be applied as follows: score 0 for a CHV who cannot perform, 1 for those who can perform but are not good, and 2 if they can perform perfectly. The rating data were ordinal, which do not provide accurate and precise measurement, that were transformed into interval data using the Rasch measurement model.

### 2.6. Data Collection and Analysis

There were 41 volunteers assessed by 20 observers using the instrument in the pre- and posttest situations. The data collected were ordinal type, which were appropriated using the Rasch model approach. First, the raw data collected were counted as frequencies implying an odd probability. After that, the probability was converted into equal-interval data using a logarithm [27, 28]. The logarithm function was used to produce measurements with the same equal-interval scale called the logit scale, which has far better accuracy and preciseness of measurement than raw data.

The Rasch model is suitable for measuring latent traits in assessing volunteers' anthropometric skills [29, 30]. Data stacking and racking were used to compare the person logit of volunteers between pre- and posttest, as well as item logit value (item difficulty level) in the two situations. The Rasch model can provide individual-centered statistics information with changing logit value of person and item. This indicates the effectiveness of CHVs' training and identifies which training part needs close attention. This is a different approach compared to regular inferential statistics, which usually only informs the situation for the entire data but cannot provide information of what is going on at the individual level.

The person logit and item logit values in the pre- and posttest situations were displayed using the Wright map, which provides the big picture of the training effectiveness. Regression analysis between item logit value in the pre- and posttest shows the changing skills that happened to each item in this study.

The psychometric attribute of the instrument in the pre- and posttest is shown in Table 1. The unidimensionality of the instrument is considered excellent if the raw variance is more than 40%, as indicated in the pretest (51.5%) and posttest (49.9%); moreover, the unexplained variance for both situations was below 15%. Reliability index for the person and Cronbach's alpha were considered good and acceptable for item reliability with good internal consistency; moreover, reliability indices did not affect the measurement quality, as the validity of instrument yielded good indices.

The item separation indicates the number of items and their difficulty levels in the instrument. The posttest index (2.17) was lower than the pretest index (4.30), and both indices were considered to be acceptable. Notably, the spread of item difficulty level was small, indicating that the training effectively increased the anthropometric skills of the volunteers.

## 3. Results and Discussion

### 3.1. Results

#### 3.1.1. Stacking Analysis

Stacking analysis was used to obtain the difference in CHVs' anthropometric skills, combining two datasets, namely, pre- and posttest assessment. By applying the Rasch model to the result of the measurement, the logit value person (LVP) was derived, which indicated how good their anthropometric skills were. A lower value, such as −1.9 logit, shows that the skills are inadequate, while higher positive logit represents CHVs' good performance. This type of analysis is called individual-centered statistics, which informs an individual CHV's situation.

The results in terms of the mean of the LVP are shown in Table 2. CHV pretest LVP mean was +0.36 logit for measuring infants (0–2 years). After the training (posttest), their LVP mean was +3.04 logit, such that the difference was 2.68 logit. The differences for measuring toddlers were even higher, 3.34 logit. Hence, training had noticeable effects in improving volunteers' anthropometric skills. The inferential statistic test for both groups was statistically significant (Table 2).

**Figure 1:**
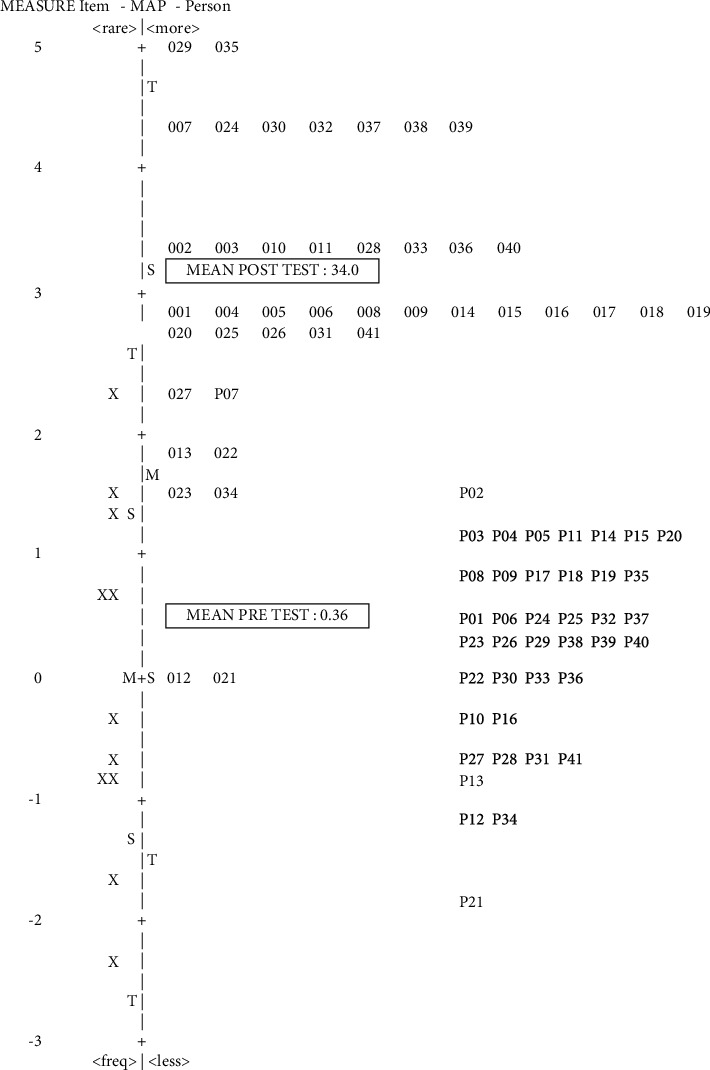
Wright map of volunteers' pretest and posttest anthropometric skills measuring infants of 0–2 years: stacking of infant practice skill.

Another result of stacking analysis is using the Wright map to provide a holistic view of the situation concerning the pretest and posttest of CHVs' LVP.  Figure 1 shows the result of the pretest (code with P) and posttest (code with 0) LVP of CHVs' anthropometric skills measuring infants. For instance, the LVP of CHV P21 in pretest was −1.8 logit and 0.0 logit after the training, showing an improvement of nearly two logit scales. CHV P28′ LVP (pretest) substantially changed to CHV 028 (posttest), showing a drastic increase in skill improvement.

**Figure 2:**
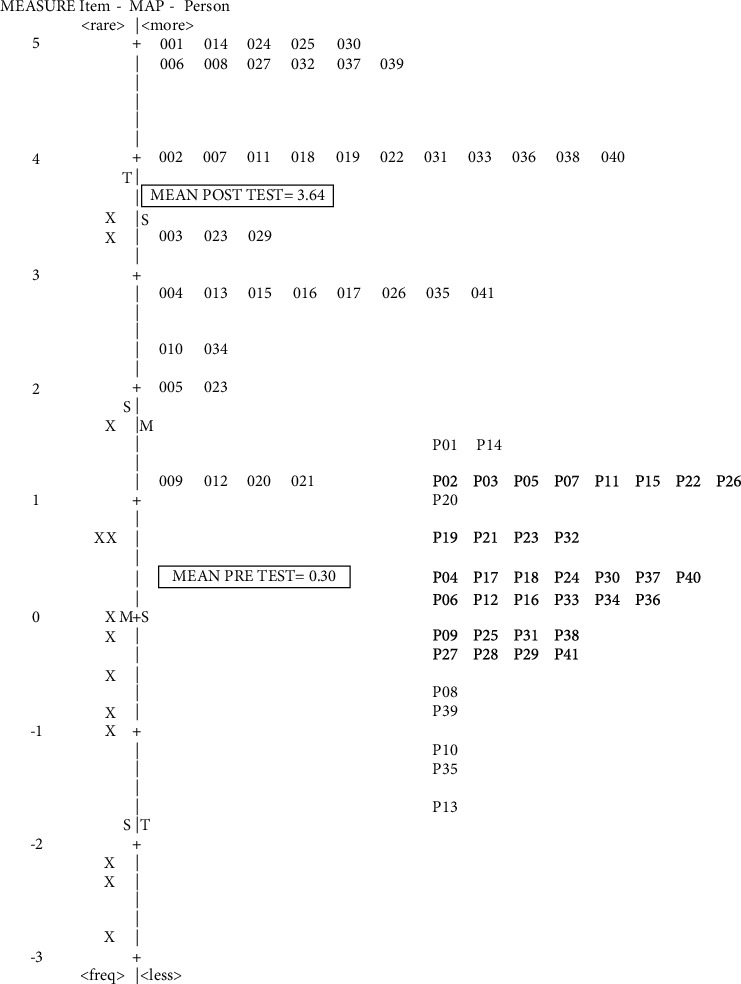
Wright map of volunteers' pretest and posttest anthropometric skills measuring toddlers (>2–5 years): stacking of toddler practice skill.

For CHVs who conducted anthropometric measuring for toddlers (>2–5 years), the Wright map showed a slightly better situation (Figure 2). Most CHVs displayed an increase in their LVP in three logit scale and mean difference between pretest and posttest by ≥3 logit. For instance, CHV P39 (pretest) logit increased by 4 logit to a posttest LVP of CHV 039. This indicates that the training indeed improved the CHVs' anthropometric skills measuring toddlers. The use of Wright's map combined with the results of the stacking method for analysis of training not only shows the ranking of CHVs' abilities but also the position of these abilities against skill indicators that are considered difficult.

#### 3.1.2. Racking Analysis

Besides the analysis to the CHVs' improvement, this study also applied analysis to the instruments, called racking analysis. In this case, racking analysis compared the difficulty level of item (logit value of item or Logit Value Item (LVI)) in the pre- and posttest situation. A decrease in LVI implies a decrease in the difficulty level. In other words, volunteers' skills improved, and they could perform the anthropometric measurement more accurately than before.

Table 3 shows the results of the LVI mean. The skills in measuring infants in pretest were +1.31 logit, and in the posttest situation, the difficulty level decreased to −1.31 logit, yielding a difference of approximately 2.61 logit scale. When measuring toddlers, the difference was even greater, that is, 3 logit scale. This shows the CHVs' skills were improving, as shown in the decreasing total mean of LVI. The inferential statistic test for both groups was statistically significant (Table 3).

**Figure 3:**
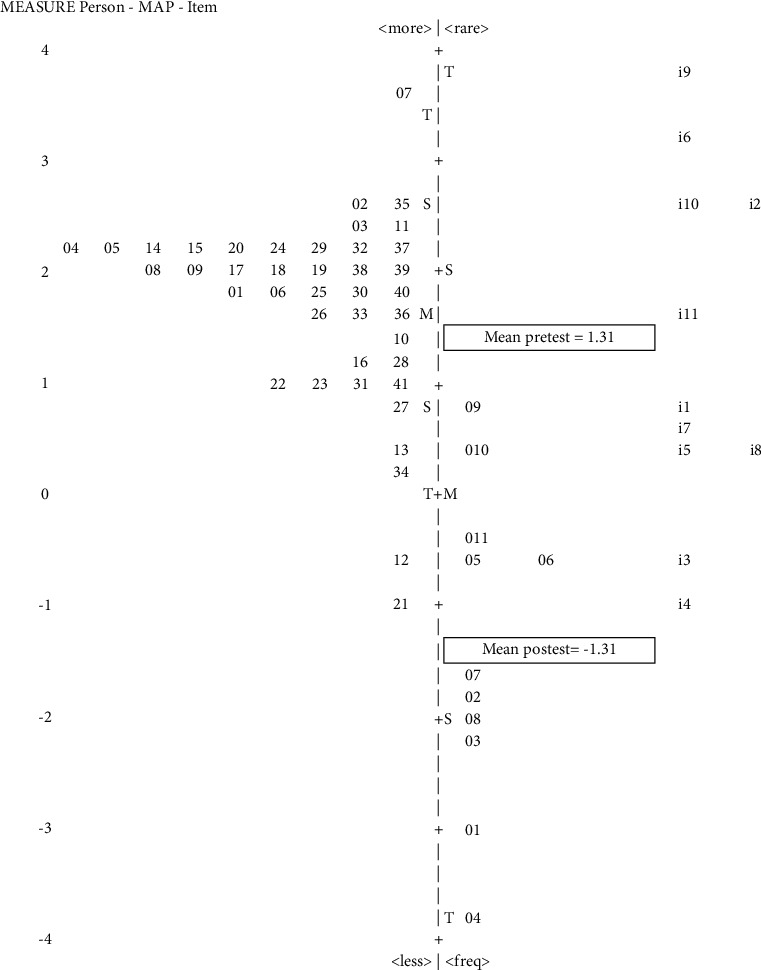
Wright map of LVI pretest and posttest anthropometric skills measuring infants of 0–2 years: racking infants practice skill.

Further analysis to acquire the details of volunteers' skill improvement was performed using the Wright map to compare LVIs in the pre- and posttest situations. The Wright map for the LVI of measuring infants showed a slightly different situation (Figure 3). Most LVI items in pretest decreased more than 2 logit scales in posttest situations. This indicates that the training certainly improved the volunteers' anthropometric skills; they became better equipped at performing anthropometric skills. Only two items were greater than 0 logit, namely, items 9 and 10, indicating that these two skills were not easy to practice.

**Figure 4:**
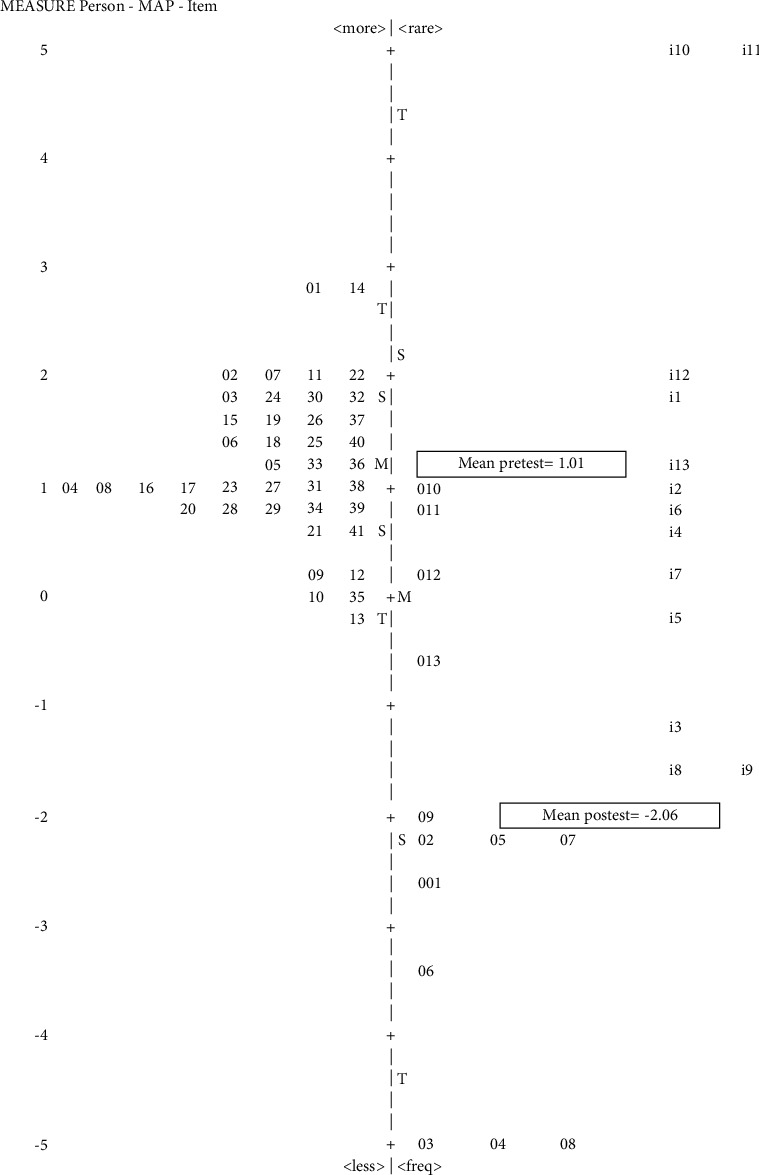
Wright map of LVI pretest and posttest anthropometric skills measuring toddlers (>2–5 years): racking of toddler practice skill.

Figure 4 illustrates the LVI changes in the pre- and posttest. For instance, item i10 (measuring shoulder and joint distances) had +5 logits in the pretest but +1 logit (code o10) in the posttest. This means that skills in i10, considered as challenging to practice by the CHV before training, could be performed better and correctly after training. In other words, the training improved the CHVs' skills for item 10, which is rarely performed in daily practice; meanwhile, improvement in skills for item 9 (recording of height measurements) was not much considering that most CHVs were already capable of adequately performing it. Three items had LVI ≥ 0.0 logit for challenging skills when measuring infants; these were items 10, 11, and 12, that is, measuring shoulder and joint distance, setting the midpoint of measurements, and measuring MUAC.

**Figure 5:**
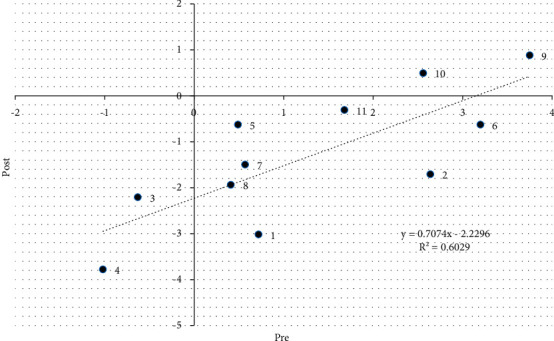
Pre-post-perception of item difficulty level of infant measurement skill.

Figure 5 illustrates the change in the level of difficulty for pre- and posttraining according to the assessment of CHVs in performing anthropometric measurements for infants (aged 0–2 years). The dotted line depicts the difficulty level, which tended to be the same for CHVs for pre- and posttest situations as assessed by observers. Items above the line represent difficult items, whereas items below the line tend to be less difficult. However, the value of the skills categorized as more difficult items did not mean that the CHVs were unable to perform them. Further details can be seen on the variable map (individual ability).

Items 9, 10, and 11 (namely, the position of measurement, determination, and documentation of head circumference measurement) were considered more difficult by the CHVs compared to other items. The village midwife should train and monitor the skill concerning head circumference measurement as CHVs have never performed head circumference measurements in anthropometric measurements.

**Figure 6:**
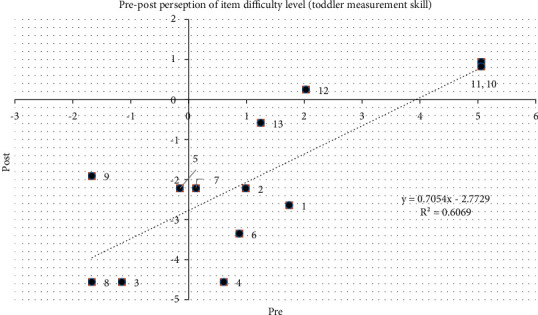
Pre-post-perception of item difficulty level of toddler measurement skill.

Figure 6 shows the change in the level of difficulty for pre- and posttraining based on the CHVs' skills in conducting anthropometric measurements in toddlers (aged 2–5 years) as assessed by observers. All items for MUAC measurement (items 10–13) were found to be more difficult by the CHVs as they had rarely performed them. To obtain these results, the trainer must perform repetitions. The use of racking analysis helps the trainer in making decisions for more precise follow-up training.

## 4. Discussions

The results revealed that CHVs' capacity increased after training. The findings that CHVs could perform appropriate measurements after being trained, although the training was short, are in line with those of the studies conducted by Mwangome et al. [20] and Moss et al. [31]. Nevertheless, there were differences between this study and that of Mwangome et al., Moss et al., and Ayele et al., [20, 31, 32]. Mwangome et al. only observed MUAC measurement with Bland–Altman and Pitman's analysis [20]. Moss et al. observed MUAC and length/height measurements using technical error of measurements (TEMs) with Bland–Altman analysis [31]. The good thing about racking and stacking analysis compared to the abovementioned methods is that the raw data transforms into equal-interval scale (logit) providing precise and accurate measurement regarding CHVs' skills (as shown in Tables 2 and 3). Furthermore, analyses with LVP (as shown Figures 1 and 2) provide individual-centered statistics that inform the big picture of skill change, whereas traditional statistics analysis rely on group-centered statistics.

Ayele et al. did not perform head circumference measurements; their analysis used and calculated several reproducibility metrics of measurements, including TEM [32]. This study assessed the results of measurements of weight, length/height, MUAC, head circumference, and stack and rack with the Rasch model. Stacking analysis can provide an overview of changes in an individual CHV's abilities after training.

Therefore, CHC, as the supervisor of IHP, should develop strategies for fostering CHVs individually and continuously, especially for those whose skills have not been improving. A systematic review conducted by Scot et al. showed that CHVs' competencies related to anthropometric skills tend to decrease after training, so regular supervision and follow-up are needed to refresh anthropometric skills [33].

Individual observations are critical because they can ensure the quality of anthropometric measurements made by CHVs. According to Anselmi et al., stacking analysis can also be used to measure changes in the perceived positive change scale from cognitive-behavioral outcome assessments to outcome evaluation [34].

Racking analysis can illustrate the changes in CHVs' skills of predetermined dimensions and indicators. Hence, the training strategy emphasizes more on aspects where CHVs have slight changes in capacity. However, the results of this study showed that MUAC measurement has a high difficulty level compared with other anthropometric measurements, even though the CHVs have been trained.

The results of this study indicated that CHVs' skills for head circumference and MUAC measurements were more challenging to perform than weight, length, or height measurements. It can be explained that head circumference and MUAC measurements for infants and toddlers are rarely performed in IHP. Hence, CHVs must be trained periodically to improve their abilities, which should be supervised by CHC. According to Tawfiq et al., regular training courses can improve the quality of the trainees [35]. de Onis et al. stated that anthropometric measurements require extensive training and supervision as well as repeated training so that the measurement results are more precise [24]. The training that has been conducted refers to the results of stacking and racking analysis. Thus, CHVs who still have inadequate capacity can meet their competence in performing anthropometric measurements.

The accuracy of anthropometric measurements in children is a critical factor in determining the quality of data on the nutritional status of children and the interventions that will be performed [19]. Significant variations in measurement results can cause measurement bias and result in a misinterpretation of the data on nutritional status in children [5].

In Indonesia, the data on anthropometric measurements performed by CHVs are used as a routine monthly data to monitor toddlers' growth. Hence, if the measurements are inaccurate, it can lead to an incorrect policy-making decision. As stated by Johnson et al., reliable child growth data are a prerequisite for monitoring and improving children's health [36]. If the measurement results are unreliable, GM cannot be reported because the problem of nutritional status cannot be detected.

The results of this study are in line with the research on the ineffectiveness of GM as a strategy for preventing and overcoming stunting. The low ability of CHVs to perform anthropometric measurements can result in inadequate identification and classification of nutritional status and diagnosis. Therefore, the capacity of CHVs must be increased in taking anthropometric measurements and their follow-up so that the GM program can run better. Additionally, CHVs can also be improved in providing counseling for mothers who have stunting babies and toddlers.

The limitation of this study is the small sample size because not all CHVs participated in this study. The trial model was limited to one district; the differences between urban and rural areas should be evaluated as they have different characteristics. The strength of this study was the use of the Rasch model technique with stacking and racking analyses that provided precise and accurate measurements; therefore, changes in the ability of each CHV and measurement items that were difficult for the CHVs could be found using these analyses.

## 5. Conclusions

The abilities of the CHVs (trainee) before training were far from adequate and were significantly improved after this training model, even though the training process was conducted in a single day. Stacking analysis facilitated trainers to observe the changes in trainees' individual abilities, whereas racking analysis facilitated the observation of changes in the perception of the difficulty level of anthropometric measurements by the trainee.

The use of racking and stacking techniques with the Rasch modeling allowed a more detailed and precise assessment. This analysis method can be used to improve the quality of anthropometric measurements performed by CHVs in conducting GM for infants and toddlers.

Inadequate GM program could lead to failure in improving nutritional status, thus limiting opportunities for children who need intervention. The capacity of CHVs in child GM must be refreshed regularly. Standardized, efficient, and proper training and its assessment should be developed to make CHV reliable in taking anthropometric measurements of infants and toddlers.

## Figures and Tables

**Table 1:** Psychometric attribute of the instrument (*N* = 41).

Psychometric attribute	Pretest	Posttest
Raw variance explained by measures	51.5	49.9
Unexplained variance	<15%	<15%
Cronbach's alpha	0.65	0.79
Person reliability	0.63	0.67
Person separation	1.3	1.42
Item reliability	0.95	0.82
Item separation	4.3	2.17

**Table 2:** Volunteers' logit value person (LVP) at pretest and posttest (*N* = 41).

	LVP pretest mean (range)	LVP posttest mean (range)	Difference	*p* ^*∗*^
Volunteers' LVP in measuring	0.36	3.04	<0.01	2.68
Toddlers 0–2 years	(−1.9 to +2.31)	(0 to +5.61)		
Volunteers' LVP in measuring	0.30	3.64	<0.01	3.34
Toddlers >2–5 years	(−1.28 to +1.57)	(+1.24 to +6.21)		

^*∗*^Wilcoxon signed-rank test.

**Table 3:** Logit value item (LVI) at pretest and posttest (*N* = 41).

	LVI pretest mean (range)	LVI posttest mean (range)	Difference	*p* ^*∗*^
LVI measuring	+1.31	−1.31	2.61	<0.01
Infants (0–2 years)	(−1.02 to 3.75)	(−3.78 to 0.88)		
LVI measuring	1.01	−2.06	3.07	<0.01
Toddlers (>2–5 years)	(−1.67 to 5.06)	(−4.56 to 0.96)		

^*∗*^Wilcoxon signed-rank test.

## Data Availability

Data used to support the study are available from the corresponding author upon request.

## Abbreviation

CHV: Community Health Volunteer
WHO: World Health Organization
GM: Growth monitoring
GMP: Growth monitoring promotion
CHC: Community health center
PPH: Public primary healthcare
MUAC: Mid-upper arm circumference
LVP: Logit value person
LVI: Logit person item
TEMs: Technical error of measurements.
